# Evaluation of twenty‐two rapid antigen detection tests in the diagnosis of Equine Influenza caused by viruses of H3N8 subtype

**DOI:** 10.1111/irv.12358

**Published:** 2016-02-01

**Authors:** Takashi Yamanaka, Manabu Nemoto, Hiroshi Bannai, Koji Tsujimura, Takashi Kondo, Tomio Matsumura, Sarah Gildea, Ann Cullinane

**Affiliations:** ^1^ Equine Research InstituteJapan Racing AssociationShimotsukeTochigiJapan; ^2^ Virology UnitIrish Equine CentreJohnstownNaasCo. KildareIreland

**Keywords:** diagnosis, equine influenza, rapid antigen detection test

## Abstract

**Background:**

Equine influenza (EI) is a highly contagious disease caused by viruses of the H3N8 subtype. The rapid diagnosis of EI is essential to reduce the disease spread. Many rapid antigen detection (RAD) tests for diagnosing human influenza are available, but their ability to diagnose EI has not been systematically evaluated.

**Objectives:**

The aim of this study was to compare the performance of 22 RAD tests in the diagnosis of EI.

**Methods:**

The 22 RAD tests were performed on fivefold serial dilutions of EI virus to determine their detection limits. The four most sensitive RAD tests (ImmunoAce Flu, BD Flu examan, Quick chaser Flu A, B and ESPLINE Influenza A&B‐N) were further evaluated using nasopharyngeal samples collected from experimentally infected and naturally infected horses. The results were compared to those obtained using molecular tests.

**Results:**

The detection limits of the 22 RAD tests varied hugely. Even the four RAD tests showing the best sensitivity were 125‐fold less sensitive than the molecular techniques. The duration of virus detection in the experimentally infected horses was shorter using the RAD tests than using the molecular techniques. The RAD tests detected between 27% and 73% of real‐time RT‐PCR‐positive samples from naturally infected horses.

**Conclusions:**

The study demonstrated the importance of choosing the right RAD tests as only three of 22 were fit for diagnosing EI. It was also indicated that even RAD tests with the highest sensitivity serve only as an adjunct to molecular tests because of the potential for false‐negative results.

## Introduction

Equine influenza (EI) is caused by equine influenza A virus (EIV) and is one of the most important respiratory diseases of horses due to its highly contagious nature.^1^, ^2^ EIV is a member of the family *Orthomyxoviridae* of the genus *Influenza virus A*.^3^ Although two subtypes of EIV have been recognized (H7N7 and H3N8), viruses of the H7N7 subtype have not been isolated for the last three decades and may be extinct.^4^ In contrast, viruses of the H3N8 subtype are a major cause of respiratory disease in horses throughout the world with the exception of the equine population in New Zealand and Iceland where EI has never been recorded.^5^, ^6^ Horses infected with EIV exhibit the acute onset of pyrexia, associated with depression and anorexia, nasal discharge and coughing.^1^, ^2^ EIV infection occurs via the inhalation of aerosolized virus particle in the same way as human seasonal influenza.^1^, ^5^

A presumptive diagnosis of EI can be made in a group of susceptible horses on the basis of rapid spread of a febrile disease with frequent coughing.^1^, ^2^ However, because the clinical severity and the rapidity of disease spread depend on the immune status of the herd, it is difficult to distinguish EI in vaccinated horses from other acute respiratory diseases, for example equine rhinopneumonitis, equine viral arteritis or equine rhinitis virus infection.^1^, ^2^ Thus, laboratory tests to identify EIV in the respiratory tract secretions or to detect seroconversion are required to make a definitive diagnosis. Virus isolation (VI), the traditional gold standard of diagnostic methods for EI,^5^, ^7^ has now been replaced by reverse transcriptase polymerase chain reaction (RT‐PCR) or real‐time RT‐PCR (rRT‐PCR) assays because of their superior rapidity and sensitivity.^8^, ^9^ Also, some rapid antigen detection (RAD) tests licensed for diagnosing human influenza have been successfully used in the diagnosis of EI.^10^, ^11^, ^12^, ^13^, ^14^, ^15^ These RAD tests enable the qualitative detection of influenza A viral nucleoprotein antigens in respiratory specimens without technical expertise and expensive equipment.^16^ Thus, they are often used as initial screening tests for EI.^10^, ^11^, ^12^, ^17^

There are a variety of RAD tests commercially available.^16^, ^18^ At the time of writing (April 2015), there were at least 21 RAD tests commercially available in Japan for diagnosing human influenza and one RAD kit for the diagnosis of avian influenza. Prior to this, no one had conducted an extensive comparison of the sensitivity of these RAD tests for the detection of EIV. We evaluated the sensitivity of selected RAD tests licensed in Japan for the diagnosis of human or avian influenza, in the detection of EIV in nasopharyngeal swabs from both experimentally and naturally infected horses.

## Materials and methods

### Viruses

EIV strain A/equine/Kildare/2/2010 was propagated in the allantoic cavity of 10‐day‐old embryonated hen's eggs. The stock virus was subjected to low‐speed centrifugation (1500 ***g*** for 10 min) to remove cell debris and was then aliquoted and stored at −80°C until use. The stock virus titre was 10^7·7^ 50% egg infectious dose (EID_50_)/ml.

### Clinical samples from experimentally infected horses

Virus challenge was carried out as previously described.^19^ Three naïve 1‐year‐old horses were inoculated by the inhalation of A/equine/Kildare/2/2010 (10^8·6^ EID_50_/8 ml/head) on Day 0. Nasopharyngeal swabs (JCB Industry, Tokyo, Japan) were collected daily from Day 0 to Day 10. Following collection, these swabs were placed in 5·0 ml of transport medium (phosphate‐buffered saline supplemented with 0·6% (w/v) tryptose phosphate broth, 500 units/ml penicillin, 500 μg/ml streptomycin and 1·25 μg/ml amphotericin B). The nasopharyngeal swabs in transport medium were subsequently vortexed for 10 second and centrifuged at 1500 ***g*** for 15 min. Supernatant was aliquoted and stored at −80°C until use. Virus isolation (VI) was conducted as previously described.^19^ The experimental protocols were approved by the Animal Care Committee of Equine Research Institute of Japan Racing Association.

### Clinical samples from naturally infected horses

Thirty rRT‐PCR‐positive nasopharyngeal samples collected during EI outbreaks in Ireland were analysed. These samples were collected from horses of mixed vaccination status and on a variety of premises including a polo yard (*n* = 6), three racing yards (*n* = 18), a non‐Thoroughbred yard (*n* = 2), a show jumping yard (*n* = 1), a Thoroughbred stud (*n* = 2) and a non‐Thoroughbred stud (*n* = 1). Following sample collection, nasopharyngeal swabs were placed in 5 ml of transport medium and rRT‐PCR was carried out as previously described.^20^ Thirty rRT‐PCR‐negative nasopharyngeal samples were also collected from racehorses in Japan in 2013.

### RAD tests

The RAD tests included in this study were purchased commercially (Table 1). These RAD tests are licensed for the diagnosis of human or avian influenza (ESPLINE A Influenza) in Japan.

**Table 1:** Comparison of the detection limits of the 22 RAD tests for equine influenza virus (A/equine/Kildare/2/2010)

Tests	Distributor	Detection limits (log EID_50_/ml)
Rapid testa FLU・NEO	Eidia Co., Ltd.	5·6
Rapid testa Color FLU stick* (OSOM Influenza A&B, Sekisui diagnostics)	Sekisui Medical Co., Ltd.	5·6
Quick navi‐Flu + RSV	Denka Seiken Co., Ltd.	5·6
Quick navi‐Flu	Denka Seiken Co., Ltd.	5·6
Prime check Flu・RSV	Alfresa Co.	6·3
Prime check Flu	Alfresa Co.	5·6
ImmunoFine FLU	Nichirei Bioscience Inc.	5·6
ImmunoAce Flu	Tauns Co.	4·9
BD Flu examan* (BD Directigen EZ Flu A+B, Beckton, Dickinson and Co.)	Beckton, Dickinson and Co.	4·9
Bright POC Flu	Shionogi Pharmaceutical Co.	5·6
Quick chaser Flu A, B	Mizuho Medy Co., Ltd.	4·9
Clearline Influenza A/B/(H1N1) 2009	Meiji Seika Pharma Co., Ltd.	>7·7
Clearview Exact Influenza A&B* (Alere Influenza A&B, Alere Inc.)	Alere Medical Co.	6·3
QUICKVUE Rapid SP influ* (QUICKVUE Influenza A&B, Quidel)	DS Pharma Biomedical Co., Ltd.	>7·7
Prorast Flu	LSI Medience Co.	5·6
POCTEM S influenza	Sysmex Co.	6·3
Capilia Flu A+B	Tauns Co.	5·6
Statmark FLU stick‐N	Nichirei Bioscience Inc.	5·6
Nanotrap Flu A・B	Rohto Pharmaceutical Co., Ltd.	5·6
Gold sign FLU	Morinaga Milk Industry Co., Ltd.	7·0
ESPLINE Influenza A&B‐N	Fujirebio Inc.	4·9
ESPLINE A Influenza	Fujirebio Inc	5·6

*These tests are available in the USA according to Website of Centers for Disease Control and Prevention (http://www.cdc.gov/flu/professionals/diagnosis/rapidclin.htm#modalIdString_CDCTable_1). The names and distributors in the USA are shown in brackets.

Testing was conducted in accordance with the manufacturers’ instructions. Initial testing to determine a detection limit for each RAD was conducted in duplicate using 100 μl of a fivefold serial dilution of the virus stock in transport medium. Further evaluation was conducted on the RAD tests with the lowest limit of detection using nasopharyngeal swabs collected from experimentally infected or naturally infected horses. All the results were judged by two operators. When the positive bands were visible by examination (approx. 7 cm from the eyes), the samples were assigned a score of + (positive). If the positive bands were not visible on the examination, the samples were deemed – (negative). In case of a judgement split, the sample was assigned +. The detection limits were expressed as the minimum virus titres (EID_50_/ml) for positive reactions.

### Viral RNA extraction

Viral RNAs were extracted from 100 μl of the stock/diluted viruses or clinical samples with MagNA Pure LC Total Nucleic Acid Isolation Test (Roche Diagnostics, Tokyo, Japan) and MagNA Pure LC 2.0 System (Roche Diagnostics) as per the manufacturer's instructions. Extracted RNA was eluted in a final volume of 100 μl.

### RT‐PCR, rRT‐PCR and RT‐LAMP

RT‐PCR with primers against the hemagglutinin (HA) gene^21^ was conducted using the Qiagen OneStep RT‐PCR kit (QIAGEN, Tokyo, Japan) as described previously.^22^

rRT‐PCR with primers and probe against the HA gene^23^ was performed using the TaqMan Fast Virus 1‐Step Master Mix (Life Technologies Japan, Tokyo, Japan) according to the manufacturer's instructions. Thermal cycling conditions were as follows: an initial hold at 50°C for 5 min, 95°C for 20 second and then 40 cycles at 95°C for 3 second and 60°C for 30 second. To quantify the copy number of EIV by the rRT‐PCR, artificial RNA was synthesized as described previously.^24^ In the initial RT‐PCR, DNA fragment of HA gene was amplified from A/equine/Ibaraki/1/2007 using the primer set described by Newton *et al*.^21^ This amplified product was then used as a template for a second PCR. The second PCR was performed using a modified forward primer (5′‐ATTAACCCTCACTAAAGGGAGAATGAGGTGACAAATGCTACTG‐3′) containing the T3 promoter sequence and the reverse primer (5′‐TGATTTGCTTTTCTGGTACA‐3′). RNA was synthesized using T3 RNA polymerase and then treated with DNase I (Roche Diagnostics). The copy number of the RNA was calculated from the absorbance value at 260 nm. A result was positive if the average copy numbers exceeded 10 RNA molecules/reaction (=5 × 10^3^ copies/ml of start sample).

RT‐loop‐mediated isothermal amplification (RT‐LAMP) was conducted using the Loopamp RNA amplification kit (Eiken Chemical, Tokyo, Japan) as previously described.^22^ These three tests were also used to determine the detection limits as mentioned above.

### Data analysis

The average detection periods were analysed by repeated‐measures analysis of variance and *post hoc* Student–Newman–Keuls method, using sigmaplot 11.2 (Systat Software, San Jose, CA, USA). A level of *P* < 0·05 was considered significant. Analysis of performance of the RAD tests with the clinical samples, sensitivity, specificity, kappa coefficient values were calculated using Excel 2010 (Microsoft Japan, Tokyo, Japan). The kappa coefficient values were evaluated according to the guideline mentioned by Landis and Koch^25^ as follows: <0 as no agreement and 0–0·20 as slight, 0·21–0·40 as fair, 0·41–0·60 as moderate, 0·61–0·80 as substantial and 0·81–1·0 as almost perfect agreement.

## Results

Using viral RNA of A/equine/Kildare/2/2010, the detection limits of the molecular tests, that is conventional RT‐PCR, rRT‐PCR and RT‐LAMP, for EIV were 10^2·8^EID_50_/ml, 10^2·1^EID_50_/ml and 10^2·8^EID_50_/ml, respectively.

The comparison of detection limits of the 22 RAD tests for EIV is shown in Table 1. The detection limits of the four tests, ImmunoAce Flu, BD Flu examan, Quick chaser Flu A, B and ESPLINE Influenza A&B‐N, were 10^4·9^EID_50_/ml. The detection limits of Prime check Flu・RSV, Clearview Exact Influenza A&B and POCTEM S influenza were 10^6·3^EID_50_/ml. The detection limit of Gold sign FLU was 10^7·0^EID_50_/ml. Clearline Influenza A/B/(H1N1) 2009 and QUICKVUE Rapid SP influ did not detect EIV in the undiluted stock virus. The detection limit of the other twelve tests was 10^5·6^EID_50_/ml.

The four most sensitive RAD tests and Prorast Flu as an example of a less‐sensitive RAD were evaluated in an EI experimental infection study, and their diagnostic sensitivity was compared to those of VI and molecular diagnostic assays. The results obtained by the different assays for the sequential samples collected from the horses experimentally infected with A/equine/Kildare/2/2010 are summarized in Table 2.

**Table 2:** Comparison of the sensitivity of virus isolation, the 3 molecular diagnostic tests and the 5 RAD tests in the detection of EI virus in three experimentally infected horses

Post‐infection day	Clinical signs*	Tests								
		Virus isolation**	Conventional RT‐PCR	rRT‐PCR***	RT‐LAMP	ImmunoAce Flu	BD Flu examan	Quick chaser Flu A, B	ESPLINE Influenza A&B‐N	Prorast Flu
0	0	0	0	0	0	0	0	0	0	0
1	0	3	3	3	3	1	0	1	0	0
2	3	3	3	3	3	3	3	3	3	3
3	3	2	3	3	3	3	3	3	3	2
4	3	3	3	3	3	3	3	3	2	0
5	3	3	3	3	3	3	2	3	2	0
6	3	2	3	3	3	2	2	2	1	1
7	3	0	1	2	2	0	0	0	0	0
8	3	0	0	1	0	0	0	0	0	0
9	3	0	0	0	0	0	0	0	0	0
10	2	0	0	0	0	0	0	0	0	0

The days of sample collection are listed in the first column. The remaining columns list the number of horses that tested positive by each assay.

*If fever (≥38·5°C) and/or nasal discharge and/or coughing was observed, the horse was scored as positive for clinical signs.

**The lowest virus titre of the virus isolation was 1·4 log EID_50_/ml.

***The lowest RNA copy number of rRT‐PCR was 5 × 10^3^ copies/ml (10 copies/test).

**Figure 1:**
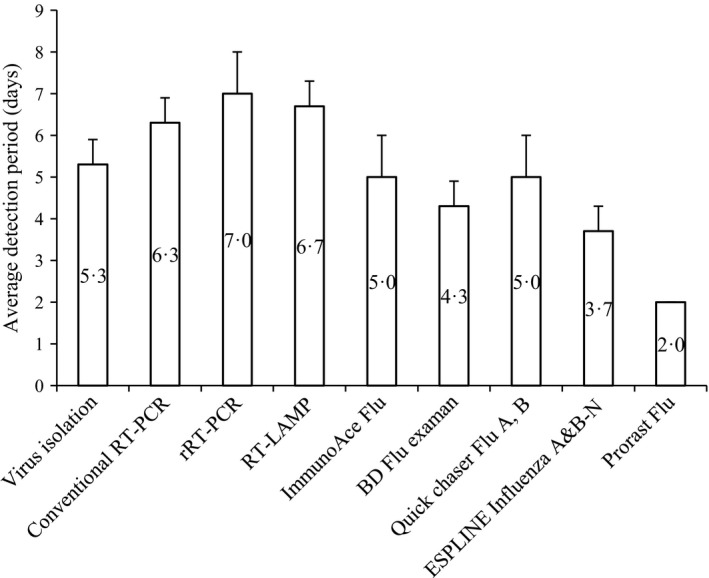
Average detection periods for the molecular and RAD tests. Vertical bars represent standard deviations.

The average detection periods for the assays are illustrated in Figure 1. The mean duration (days) of positive rRT‐PCR results was significantly longer than those of all the RAD tests (*P* < 0·001 to =0·034). The mean duration of RT‐LAMP‐positive results was significantly longer than those of the RAD tests (*P* < 0·001 to =0·011), except for ImmunoAce Flu (*P* = 0·05) and Quick chaser Flu A, B (*P* = 0·073). The mean duration of conventional RT‐PCR‐positive results was significantly longer than those of the RAD tests (*P* < 0·001 to =0·025), except for ImmunoAce Flu (*P* = 0·087) and Quick chaser Flu A, B (*P* = 0·143). The mean duration of positive Prorast Flu results was significantly shorter than those of all the other tests in this study (*P* < 0·001 or 0·011). There was no significant difference in the mean durations of positive results obtained with the molecular assays (*P* = 0·502–0·575).

The evaluation of the performances of the most sensitive RAD tests to diagnose EI in field samples is summarized in Table 3. All rRT‐PCR‐negative nasopharyngeal samples were negative by the RAD tests. Of the 30 rRT‐PCR‐positive samples, BD Flu examan detected 22 (73%) and Quick chaser Flu A, B and ImmunoAce Flu detected 20 (67%) as positive. ESPLINE Influenza A&B‐N was less sensitive and detected only eight positive samples (27%). Kappa coefficient values indicated a substantial agreement between BD Flu examan, Quick chaser Flu A, B and ImmunoAce Flu and rRT‐PCR but only a fair agreement between ESPLINE Influenza A&B‐N and rRT‐PCR.

**Table 3:** Performances of 4 RAD tests in the detection of EI virus in 30 rRT‐PCR‐positive samples collected from infected horses in Ireland and 30 rRT‐PCR‐negative samples collected from horses in Japan

RAD tests	Test results		Sensitivity (95% confidence interval)	Specificity (95% confidence interval)	Kappa coefficient value
	Positive	Negative			
ImmunoAce Flu	20	40	67% (47, 83)	100 (88, 100)	0·67
BD Flu examan	22	38	73% (54, 88)	100 (88, 100)	0·73
Quick chaser Flu A, B	20	40	67% (47, 83)	100 (88, 100)	0·67
ESPLINE Influenza A&B‐N	8	52	27% (12, 46)	100 (88, 100)	0·27

## Discussion

Rapid diagnosis and isolation of horses with EIV are the most effective measures for the reduction in disease spread during an outbreak.^1^, ^2^ The isolation of the RAD‐positive horses played a key role in minimizing the spread of EI during the outbreak in 2007 in Japan.^26^ The RAD tests were conducted using ESPLINE Influenza A&B‐N on the basis of previous reports which indicated a comparable sensitivity for ESPLINE Influenza A&B‐N and BD Directigen Flu A+B in the detection of EIV.^15^ However, ESPLINE Influenza A&B‐N was modified in 2013, and BD Directigen Flu A+B was replaced by the new product, BD Flu examan in Japan or BD Directigen EZ Flu A+B in other countries.^16^, ^18^, ^27^ To the best of our knowledge, this study is the first comprehensive evaluation of RAD tests currently on the market.

Sakai‐Tagawa *et al*.^18^ reported that there are 100‐fold differences in sensitivities among RAD tests in the detection of avian H5N1 or H1N1pdm. Likewise, in this study, the detection limits of 20 of the 22 RAD tests evaluated varied up to 125‐fold and two did not detect EIV in undiluted stock virus of 10^7·7^EID_50_/ml. Only four RAD tests (ImmunoAce Flu, BD Flu examan, Quick chaser Flu A, B and ESPLINE Influenza A&B‐N) commercially available were able to detect EIV at 10^4·9^EID_50_/ml. This indicates the importance of selecting a suitable RAD test when screening for EI, a finding supported by the results of the experimental infection study summarized in Table 2. In that study, a fivefold difference in detection limits between Prorast Flu and the other four RAD tests, ImmunoAce Flu, BD Flu examan, Quick chaser Flu A, B and ESPLINE Influenza A&B‐N, resulted in false‐negative results with Prorast Flu and a failure to detect EI in the majority of positive nasopharyngeal samples especially on Days 4 and 5. ImmunoAce Flu, BD Flu examan, Quick chaser Flu A, B and ESPLINE Influenza A&B‐N performed well, indicating the usefulness of these RADs in the diagnosis of EI in immunologically naïve horses. They typically missed the detection of virus on the first day when all three horses were positive by the molecular tests and virus isolation. However, at this point in time, the horses were showing no clinical signs; therefore if testing due to suspicion of EI, samples would not have been collected on that day. The first day of clinical signs (Day 2) was associated with all four RAD tests successfully detecting virus in all horses. Day 6 was the last day of RAD test positivity, and samples were also culture negative after Day 6. If a RAD test was used to confirm a clinical diagnosis in such horses, it is likely that the traditional guideline of 1 week of complete rest for every day of increased temperature^5^ would be followed and the horses isolated from other horses. In such a case, the fact that the RAD tests were less sensitive than the rRT‐PCR which detected virus up to Day 8 would not have a major practical implication. Furthermore, there was no difference in specificity between the four RAD tests, indicating the low risk of false‐positive results.

Although ImmunoAce Flu, BD Flu examan, Quick chaser Flu A, B and ESPLINE Influenza A&B‐N showed the highest sensitivity with the detection limit at 10^4·9^EID_50_/ml among the 22 RAD tests, their sensitivities were 125‐fold lower than those of the molecular diagnoses (10^2·1^ to 10^2·8^EID_50_/ml). These large differences in the sensitivity between the RAD tests and the molecular tests indicate that the latter are the most appropriate tests for EI diagnosis. However, ESPLINE Influenza A&B‐N and BD Directigen Flu A (or A+B) were used for the screening of horses in quarantine stations and the identification of infected horses during EI outbreaks.^10^, ^11^, ^12^, ^17^ Sensitive RAD tests may serve as an adjunct to molecular tests, and positive results are instructive as the specificities of the tests. They are a rapid and easy way to carry out preliminary screening if EI is suspected but are best used in a population of horses rather than in individual cases. Negative test results need to be verified by a molecular test; thus, the RAD tests are unsuitable for testing for freedom of infection prior to movement.

Over the decades, the rapid international movement of horses by air has increased the risk of virus incursions into susceptible population.^28^, ^29^ Therefore, the detection of EIV‐positive horses in quarantine facilities is an important control measure and the tests need to be of optimum sensitivity. The majority of horses are immunized between 21 and 90 days before shipment either with a primary course or with a booster. Thus, they are likely to shed far less virus than the horses in the experimental study discussed above and may show few or no clinical signs. Furthermore, quarantine periods vary depending on the importing country, the purpose of movement and the status of the exporting country. Thus, the window of opportunity for the detection of EI may be quite short and the nasal swabs may be collected prior to or after virus shedding has peaked. Our results suggest that the molecular diagnosis for EIV is more likely to protect susceptible local populations from the intrusion of EIV than a reliance on RAD tests which are less sensitive. Of the molecular tests used in this study, rRT‐PCR appeared to be the most sensitive assay with a detection limit of 10^2·1^EID_50_/ml, which can be converted to approx. 10^−0·6^EID_50_/2 μl/reaction, assuming that the efficiency of RNA extraction is 100%. The RAD tests enable veterinarians and others responsible for the health of horses to perform the rapid diagnosis of EI without special equipment and technical expertise. However, the usage of even the most sensitive RAD tests is best limited to population screening and all negative samples from suspect cases should be submitted to a laboratory for further testing.

In summary, the results of this study indicate that the sensitivities of the 22 RAD tests commercially available in Japan in the detection of EIV vary hugely. Moreover, this study showed that only three of 22 RAD tests, namely ImmunoAce Flu, BD Flu examan and Quick chaser Flu A, B, were substantially fit for diagnosing EI. As the viral nucleoprotein is highly conserved for the last three decades (>98% amino acid homology, data not shown) in EIV (H3N8), sequence diversity should not affect the sensitivity of these RAD tests. However, because these tests are frequently modified and their availability varies from country to country, re‐evaluations of their performances may be periodically required in each country. RAD tests are not necessarily performed by equine healthcare professionals, and it is essential that those responsible for screening horses are made aware that the majority of such tests are unsuitable for the detection of EI‐infected horses. It is imperative to select an appropriate RAD and to submit negative samples from suspect cases to a laboratory for confirmatory testing.

## Competing interests

The authors declare that they have no competing interests.
